# Factors Influencing Children’s Eating Behaviours

**DOI:** 10.3390/nu10060706

**Published:** 2018-05-31

**Authors:** Silvia Scaglioni, Valentina De Cosmi, Valentina Ciappolino, Fabio Parazzini, Paolo Brambilla, Carlo Agostoni

**Affiliations:** 1 Fondazione De Marchi—Department of Pediatrics, IRCCS Ca’ Granda Ospedale Maggiore Policlinico, 20122 Milan, Italy; sscaglioni50@gmail.com; 2 Pediatric Intermediate Care Unit, Fondazione IRCCS Ca’ Granda Ospedale Maggiore Policlinico, 20122 Milan, Italy; valentina.decosmi@unimi.it; 3 Branch of Medical Statistics, Biometry, and Epidemiology “G. A. Maccacaro”, Department of Clinical Sciences and Community Health, University of Milan, 20122 Milan, Italy; 4 Department of Neurosciences and Mental Health, Fondazione IRCCS Ca’ Granda Ospedale Maggiore Policlinico, University of Milan, 20122 Milan, Italy; valentina.ciappolino@libero.it; 5 Department of Clinical Sciences and Community Health (DISCCO), University of Milan, 20122 Milan, Italy; fabio.parazzini@unimi.it; 6 Department of Obstetrics, Gynecology, and Neonatology, University of Milan, Fondazione IRCCS Ca’ Granda Ospedale Maggiore Policlinico, Via Commenda 12, 20122 Milan, Italy; 7 Department of Pathophysiology and Transplantation, University of Milan, 20122 Milan, Italy; paolo.brambilla1@unimi.it; 8 IRCCS “E. Medea”, Bosisio Parini, 23900 Lecco, Italy

**Keywords:** parental influences, obesogenic environment, family meal, child food preferences, taste

## Abstract

Relevant factors involved in the creation of some children’s food preferences and eating behaviours have been examined in order to highlight the topic and give paediatricians practical instruments to understand the background behind eating behaviour and to manage children’s nutrition for preventive purposes. Electronic databases were searched to locate and appraise relevant studies. We carried out a search to identify papers published in English on factors that influence children’s feeding behaviours. The family system that surrounds a child’s domestic life will have an active role in establishing and promoting behaviours that will persist throughout his or her life. Early-life experiences with various tastes and flavours have a role in promoting healthy eating in future life. The nature of a narrative review makes it difficult to integrate complex interactions when large sets of studies are involved. In the current analysis, parental food habits and feeding strategies are the most dominant determinants of a child’s eating behaviour and food choices. Parents should expose their offspring to a range of good food choices while acting as positive role models. Prevention programmes should be addressed to them, taking into account socioeconomic aspects and education.

## 1. Introduction

Food provides nutrients and gives energy. Nutrients are essential for human health, but also other compounds continue to be identified in foods, and their health properties are becoming better understood [1]. The correlation between nutrients, foods, and dietary patterns has important implications, especially for prevention and development of chronic diseases, such as cardiovascular diseases (like heart attacks and stroke), cancers, chronic respiratory diseases (such as chronic obstructive pulmonary disease and asthma) and diabetes [2]. Food preferences continue changing throughout life, under the influence of biological, social, and environmental factors [3]; these preferences are key determinants of food choices, and therefore diet quality [4,5].

Following an ecological model of developing food choices, we should consider the pioneering theory of Urie Bronfenbrenner, since it has the potential to influence new directions and development in Child and Youth Care. This Ecological Systems Theory states that human behaviour depends on the interaction of different environmental factors and personal characteristics, such as genetics, gender, and age [6].

The child’s ecological niche includes family and peers, which are both influenced by community, society, media, and food offering. Variety and complexity of children’s milieu increases throughout life [7]. Parents provide food environments and experiences with food and eating for their children. Children model themselves on their parents’ eating behaviours, lifestyle, eating-related attitudes, and satisfaction or dissatisfaction regarding body image.

Dietary habits are shaped at a young age and maintained during later life with tracking over time [8]. Eating behaviours established in childhood persist, with implications such as fussiness and poor dietary variety, or high responsiveness to food cues and increased obesity risk. Although eating behaviours and child weight are difficult to modify directly, parental feeding practices are potentially a good target for interventions to prevent unhealthy eating patterns and developing excess weight in children [9].

**Figure 1:**
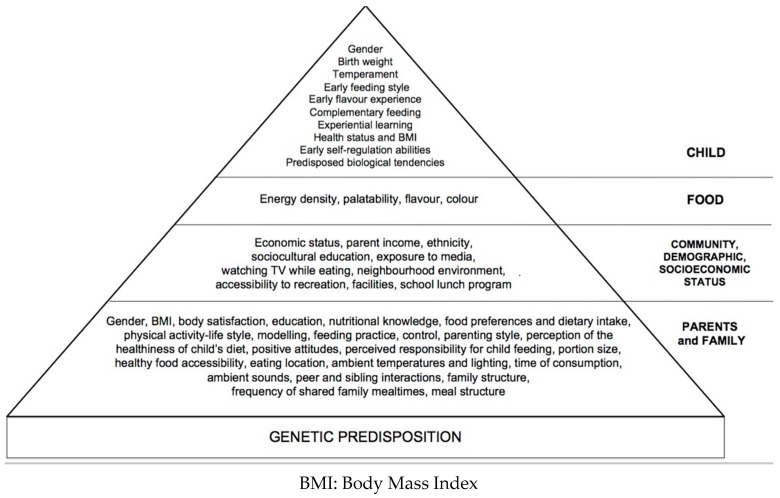
Factors influencing children’s eating behaviours from [11].

Studies into determinants of human eating behaviours have examined separate elements with the risk of not understanding the real contribution of each factor. Our narrative review describes family environment with an emphasis on parental role and strategies to improve children’s eating behaviours; highlights early feeding experiences and later food choices; describes obesogenic environments, in particular, media inputs, as well as socioeconomic and educational status. Relevant topics will be discussed and updated from previous articles [10]. Knowledge of mechanisms underlying food habits may be helpful to paediatricians to favour the creation of healthy food practices throughout the population of children. The study of children’s behaviour should thus be seen as a starting point for targeted and effective nutrition education programmes, while at the same time suggesting further research strategies to elucidate the interactions between the various factors influencing children’s eating behaviours (Figure 1).

## 2. Methods—Literature Search Strategy

Electronic databases (PubMed, Medline, Embase, and Google Scholar) were searched to locate and appraise relevant studies. We carried out a search to identify articles of potential interest published in English on factors that influence children’s feeding behaviours. Relevant articles published from 2011 to January 2018 were identified using the following groups of key words.

Articles of potential interest were selected for inclusion in this narrative review if they addressed one of the following pathways, nominated due to previously reported associations between: (A) children eating behaviors AND parental feeding practices; (B) children eating behaviors AND family eating environments; (C) family eating environments AND children choices; (D) children eating behaviors AND family meals; (E) children eating behaviors AND parental influences; (F) children eating behaviors AND obesogenic environment; (G) parental modelling of eating behaviours; (H) children eating behaviours and socioeconomic status. Child eating was defined as dietary intake, diet patterns, intakes of specific foods or beverages, food choices, food preferences, eating styles and eating behaviours. Parenting behaviours included specific feeding behaviours (e.g., using food as a reward, modelling) and general parenting behaviours. All studies had to provide measures of anthropometric status.

**Figure 2:**
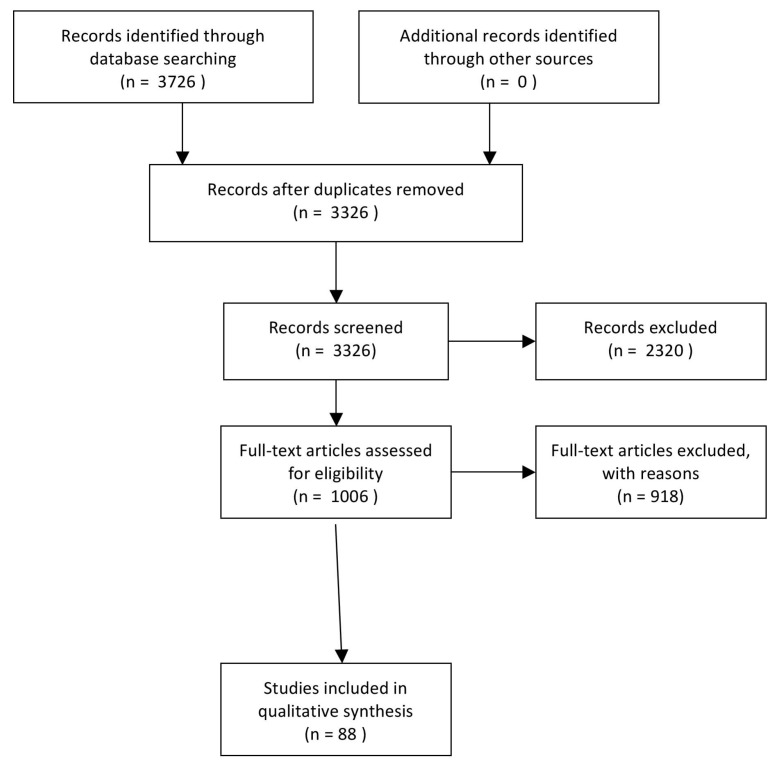
PRISMA Diagram of search strategy.

To be included, studies needed to focus on high socio-economic or Industrialized groups or with the overall results stratified by socioeconomic or Industrialized group, or to report on interactions between socioeconomic or Industrialized group and the pathway variables. Socioeconomic ad-vantage was defined based on families being described as having high income, high level of education or occupation, and/or living in an area defined as advantaged using aggregate indicators. 3726 references matched the terms of the search. The author excluded 2910 articles and assessed the potentially relevant ones following these inclusion and exclusion criteria. As our focus was from infancy to adolescence, studies of children ages 6 months through 19 years were included. Studies focusing on weight loss or with children with underlying medical conditions were excluded. No limitations were placed on publication year, although studies needed to be published in English and use human participants. However, we limited our search to industrialized countries as our focus was on the effects in high-income countries. Studies included had to be primary studies or papers presenting secondary data analyses from these studies and be published in a peer-reviewed journal or edited book. The full search strategy is reported in Figure 2.

## 3. Family Environment

The importance of the family environment for children’s and adolescents’ health behaviours has been demonstrated, but the underlying mechanisms of this influence remain unclear [12]. Previous studies have indicated that a positive family system may be part of a process that establishes and promotes beneficial health behaviours through role modelling, provision of healthy foods, and support for engaging in healthy eating behaviours [13].

The family can be considered a system, as it is more than the sum of individuals. One relevant aspect of the family environment may be the “family health climate” [14], which is defined as the shared perceptions and cognition concerning a healthy lifestyle within a family. It reflects the individual experience of daily family life, the evaluation of health-related topics, and expectations with respect to typical values, behaviour routines, and interaction patterns within the family. This conceptual framework includes psychosocial concepts such as family functioning, cohesion, conflicts, communication, socioeconomic status, parental practices, and parental style. Children’s ability of imitating the actions of the others and learning by observation in particular from their parents’ and caregivers’ could explain the kind of food styles developed [15].

## 4. General Parental Influences

Studies on the influence of parenting on child outcomes have relied on four parental prototypes, that were developed by Baumrind almost four decades ago [16]. Initially Baumrind identified only three parenting styles that were classified as: Authoritative, Permissive, and Authoritarian Parenting styles. In 1983, in a review of Baumrind’s work, Maccoby and Martin updated her parenting styles and added a fourth: Uninvolved or Neglectful [17].

Authoritative parents are demanding and responsive and are characterized by high levels of control and warmth; they monitor the child’s behaviour and convey clear standards without resorting to intrusive or restrictive approaches. Authoritarian parents are demanding and directive with low levels of responsiveness; they exhibit high levels of control [similar to authoritative parents], but in contrast show lower levels of warmth. Permissive parents are less likely to be demanding and to require mature behaviour but exhibit high levels of responsiveness; they tend to be lenient and avoid confrontation. Rejecting/neglecting parents are neither demanding nor responsive.

Using this construct, children exposed to authoritative parenting show the highest levels of self-efficacy, self-discipline, emotional maturity and improved their eating behaviours [18,19]. Indeed, studies have showed how an authoritative parenting style also is associated with a lower risk for obesity [20]. Furthermore, parent feeding behaviour is itself influenced by many factors including peers, more than by dietary guidelines [21]. Food preferences are important determinants of children’s food intakes. Parental feeding behaviours have a significant influence on the development of children’s food preferences. To affect their child’s food preferences, parents utilize many diverse behaviours that are a mixture of effective and ineffective strategies [22]. They influence how children’s intake patterns are set, both directly and indirectly, adopting overt and covert control. Overt control includes both restriction and pressure to eat. Covert control includes strategies such as purchasing only healthy foods for the home and avoiding stores and restaurants that sell unhealthy foods. The child can detect overt control, but not recognize covert control. Birch et al. [22] provided the first experimental evidence that parents’ use of restrictive feeding practices is counterproductive; it increases preschool children’s intake of restricted foods and is a risk factor for excessive weight gain. Pressure to eat was associated with higher food avoidance traits and lower consumption of core foods. Monitoring practices were related to lower food avoidance and food approach traits and lower non-core food consumption [15]. Recently, Rollins et al. [23] confirmed that restrictive feeding practices are counterproductive, and children with lower self-regulation and at risk for obesity show greater susceptibility to the negative effects of restrictive feeding. Nevertheless, the same authors [23] concluded that, in the current obesogenic environment, some parental control is likely required to moderate children’s intake of these foods. This finding reinforces the hypothesis that an authoritative parenting style, in which parents use moderate levels of control, may facilitate the development of children’s self-regulation and moderate children’s intake of palatable snack foods, promote children’s diet quality, and reduce obesity risk [24].

## 5. Maternal Influences

Mothers are often responsible for determining how much food is offered to their children. However, the factors that influence a mother’s decisions as to how much to offer her children, and her motivations and goals for feeding and consumption are poorly understood. According to recent research findings, mothers have emotional investments in their children’s eating, and portion sizes offered differ for children who are “good” eaters and “picky” eaters. Some influencing factors were child-centred (e.g., the child’s likes and dislikes and foods previously eaten in the day) and some related to adult expectations and concerns, in particular, nutrient content and waste. Mothers know the “right amounts” to serve their child and have emotional investments in their children’s eating. Interventions focusing on portion size may be more effective if tailored to the mothers’ perception regarding [25,26]. Furthermore, Bouhlal et al. demonstrated that child gender may influence mothers’ food choices, as the caloric content of boys’ meals was higher than girls’ and this extra caloric difference was from the less healthy food category [27].

Bergmeier et al. studied associations between reported and observed maternal pressure to eat and factors influencing mothers’ control [28]. The comparison between reported and observed maternal pressure to eat towards infants, shows that some mothers are not aware of their practices. Maternal pressuring or restricting the consumption of a particular food was linked to concerns about weight and the child’s propensity to gain excess weight. As their children’s age increases, parents’ awareness about food and eating changes. Over time, parents may gain confidence in their child’s ability to respond to natural satiety cues or they may develop other strategies, such as using covert methods to limit access to foods they want their children to avoid [22]. To investigate associations between parental modelling with healthy and unhealthy food intake in mothers and children, a specific questionnaire has been developed. It takes into account verbal modelling and unintentional modelling for cases in which children adopt eating behaviours that parents have not actively modelled [29]. These studies in toddlers and preschool children suggest that mothers may intentionally model healthy food intake while unintentionally acting as role models for their children’s less healthy snack food intake. Mothers also influence children directly during mealtimes. Mothers also influence children directly during mealtimes; mothers of obese children may alter their feeding behaviour differentially based on food type [30]. Maternal actions also act indirectly by shaping the behaviour of siblings that may act as caregivers and role models. This association between maternal feeding behaviours and encouragements to eat de-rived from sibling to the index child during mealtimes was shown by Mosli in a group of 69 children aged 4–8 years [31].

## 6. Paternal Influences

Fathers have a great deal of influence on young children’s nutrition and some differences were noted when compared to mothers’ feeding practices. Fathers are generally less likely to monitor children’s food intake and to limit access to food. The common feeding influence is pressuring children to eat [32]. Khandpur et al. [33] showed that use of excessive control over a child’s feeding disregards the child’s independence. On the contrary, being indulgent to a child’s food requests is also inappropriate, in that it may override a child’s ability to eat according to internal hunger and satiety cues. Both these behaviours may lead to overeating and may lead to excess weight gain. On the other hand, responsive feeding practices involve identifying and appropriately responding to the child’s satiety and hunger cues [9].

The majority of the feeding practices studied by Khandpur et al. were responsive and included encouragement or support of the child’s autonomy and independence, moreover they help in organizing the feeding environment to improve the child’s competence in choosing and eating meals [33]. Guerrero et al. [34] investigated the frequency of out of home meals with fathers and reported that these eating activities were associated with consumption of fast foods and artificially sweetened beverages by the children. In addition, they found that when fathers ate breakfast with their children, sweetened beverage consumption decreases.

Despite their expanding role in child rearing, fathers are under-represented in child feeding research. Available studies provide evidence that fathers’ eating behaviours are potentially modifiable and may be critical components of paediatric weight management interventions, both in clinical and in community settings [34].

## 7. Family Meals

Individual interactions affect the family environment. The physical characteristics of the home environment include the accessibility and availability of different foodstuffs, while family meals represent the key sociocultural setting. By the way mealtimes offer a naturalistic setting where parents are often managing child behaviours, imposing rules and expectations, and interacting with their children. For these reasons family meals and social interactions during the meals are important events in a child’s life [35] and are linked to the child’s weight status and to the development of his or her eating patterns; relations between frequency of family meals and nutrient intake, food intake, obesity, disturbed/disordered eating practices, and psychosocial effects exist in all age groups [36].

Dietary quality is influenced by practices such as eating breakfast [37], family meals [38], and fast-food consumption [39]. Both dietary quality and meal practices are linked with sociodemographic characteristics [40,41,42].

Adolescents and children who join in fewer family meals consume more unhealthy food. Indeed, there is a positive relation between frequent family meals and greater consumption of healthy foods (i.e., fruits, vegetables, and calcium-rich foodstuffs) [43].

Nutrient and caloric intake at family meals depends on the foods served and providing fast food and takeout food items at family meals may negate the nutritional benefits usually associated with home-cooked family meals [44].

A study of 2–5-year-old children in the United Kingdom found that eating the same food as their parents was the best predictor of pre-schooler vegetable consumption. Accordingly, fruit, vegetable and whole grain intake frequencies were associated positively with vegetable availability, family meals, and breakfast, and inversely with fast food. Fizzy drinks and snacks were positively associated with television meals and fast food, whereas fizzy drinks were inversely associated with breakfast frequency [44]. The benefits of family meals track into the teen and young adult years. Sharing breakfast with parents when a child is 10 years old is associated with a higher probability that child will have more frequent breakfasts when he or she is 16. Young adults who ate daily family meals during adolescence ate more servings of fruits and vegetables daily as young adults than peers who never shared family meals in adolescence [45].

Parents also consider family meals to be an opportunity to increase interaction with their offspring and share their values that are associated with food and eating. Watching television during family meals appears to negate benefits associated with frequent shared meals [46].

Individuals may be more likely to overeat when watching television and may learn unhealthy food habits from advertisements and programmes [47]. Children who watch television during two or more meals per day consume fewer servings of healthy food and more red/processed meat and junk food than children from families in which television was never on during mealtimes or only for one meal per day [46,47]. The IDEFICS study (“IDentification and prevention of dietary and lifestyle induced health Effects in Children and InfantS”) was a survey conducted in eight European countries on 15,144 children from 2 to 9 years old. Researchers assessed the children’s anthropometry and administered questionnaires to the parents regarding their children’s diets and television habits. A subsample of 1696 schoolchildren underwent further sensory testing for fat and sweet taste preferences and usual consumption of foods high in fat and sugar. All television indicators were significantly associated with increased risk of being overweight. School children with more television exposure consume high sugar and high fat foods without having a preference for sweet- or fat-enhanced test foods, suggesting that passive consumption of these products may be occurring in association with television [48]. Possible biases derive from the fact that the parents were reporting the television viewing and dietary data, so that the data may be imprecise and also be influenced by a desire to report healthy habits. Fitzpatrick et al. [49] reported that, in households where the television was on during family meals, the odds of serving vegetables and fruits at meals at least twice a day decreased significantly. Finally, teens who more frequently share family meals appear to engage in fewer risk-taking behaviours, including drinking alcohol and using illegal substances, so families’ meals can be considered as part of childhood obesity prevention strategies [50].

In conclusion, clinicians should advise their patients about the benefits of sharing three or more family mealtimes per week; benefits include a reduction in overweight, eating unhealthy foods, and disordered eating, as well as an increase in the consumption of healthy foods [51].

## 8. Education and Socioeconomic Status

In the developed world, obesity is closely connected with low socioeconomic status (SES), which, in turn is a strong determinant of the dietary intake of children and adolescents [52]. Social groups in which children are embedded transpose their social norms and attitudes and act as “communication buffers” between them and media messages that group members filter and evaluate. There is an association between maternal educational level and healthy eating behaviour in children and adolescents. In USA, infants of mothers who have low levels of education, or who are of non-Hispanic African American extraction (versus non-Hispanic Caucasian) have a higher intake of sugar, fat, and protein, with greater increase in body mass index (BMI) *z* scores from age 6 to 12 months [45]. Children of mothers with a high educational level consumed more fruit and vegetables and were more likely to have breakfast on a daily basis than children of mothers with a low educational level [53].

In an analysis of prospective data from the New England Family Study on 565 subjects (a 2005–2007 adult follow-up of a cohort initiated in 1959–1966) found that the effects of SES are long lasting [54]. Childhood social environment (at age 7 years) was assessed using a cumulative index of socioeconomic and family stability factors, built on 10 binary factors measured with a questionnaire given to mothers between pregnancy and age 7 years. The results demonstrated that social disadvantage in childhood may contribute to the development of cardio metabolic disease in adulthood by predisposing the children to adopt unhealthy behaviours (particularly cigarette smoking and excess drinking). These effects may be manifest more than 40 years later and lead to higher BMI, over and above the influence of adulthood SES [54,55].

The study of Kim et al. [56] examined the trend in unhealthy food intake among Korean adolescents by socioeconomic position. They found that there was a positive effect of nutritional policy on unhealthy food intake, and the group with high socioeconomic status appeared to undergo greater salutary changes in dietary behaviours after implementation of nutritional policies than the low socioeconomic group. Food preferences also have been found to be related to parental feeding behaviours, economic conditions, and knowledge of nutrition in Chinese children [57].

## 9. Child Eating Behaviour

Parents are influenced, in turn, by their children’s behaviours and characteristics. Parents of preschool children have been found to adapt their controlling feeding practices in response to their child’s weight: they tend to pressure infants who are lighter and have a smaller appetite, and restrict infants with larger appetites, in particular, if they are bottle-fed [58]. Similar findings have been reported for appetitive behaviour, with parents exerting more pressure on a child who shows less interest in food and being more restrictive with a child who is very food responsive [59]. The use of food as a reward and restriction of food for health reasons with 3–5-year-old children may be responsible for a high intake of food during periods of negative emotion at 5–7 years of age [60].

Excessive control of 5–7-year-old children’s food intake may unintentionally teach children to eat palatable foods to manage negative emotions. The effects of restriction, then, differ by children’s regulatory and appetitive tendencies. Greater increases of intake in response to restriction were observed among children with lower inhibitory control who found the restricted food highly reinforcing and who had previous experience with parental use of restriction [61].

## 10. Food Preferences

Recognition of tastes and odours develops before birth during foetal development, as the foetus swallows amniotic fluid, which is flavoured by the mother’s diet, including aromatic compounds such as garlic, anise, and onion. There is considerable interest in prenatal programming of taste preferences as it may influence early acceptance of nutritious foods [62].

Early in life, most infants and children prefer sweet and salty flavours. Sweetness is a potent psychobiological stimulus for many animal species, particularly for humans of all ages. Sweetness clearly increases the palatability of foods and beverages, stimulating intake [63]. Bitter flavours, such as those in some vegetables, are often rejected when first experienced, but accepted with increased exposure. Perception of taste may be varying between individuals depending on variations in taste receptors genes [64]. After birth, breast-fed infants are still exposed to flavours from the maternal diet. In contrast, formula-fed infants learn to prefer its unique flavour profile and may accept, later on, a varied diet with more difficulty [64]. Regardless of early feeding mode, infants can learn through repeated exposure and dietary variety if caregivers focus on the child’s willingness to consume a food and not just the facial expressions made during feeding. Introduction to a variety of fruits and vegetables and limiting non-core food exposure from an early age are important strategies to improve later diet quality. Hetherington et al. conducted a randomized intervention study on intake and liking of vegetables with 36 mothers and infants. They tested a step-by-step exposure to vegetables, first in milk, and then in rice, during complementary feeding periods. They concluded that early exposure to vegetables in a step-by-step protocol could be successfully introduced in complementary feeding guidelines [65].

In some cultures, children are deliberately exposed to strong flavours. For example, in Mexico, they are given food flavoured with chilli peppers at gradually increasing strength. Learning to like initially unpalatable foods may be part of a process of socialization. Individual patterns of food preferences and eating behaviours emerge and differ, depending on the foods offered and on the contexts of feeding during the complementary feeding. Infants who were previously exposed to a greater variety of solid foods show fewer rejection behaviours in response to later offers of novel foods [66]. Food neophobia, the predisposition for rejecting unfamiliar or unknown foods, is a normal developmental phase, that typically peaks between 2 and 6 years of age. Children who are more neophobic tend to most commonly show lower preference for vegetables [67]. The related construct of food “fussiness” [or “pickiness”] has also been linked with lower dietary variety and quality. Fussier children, in addition to refusing new foods, often eat a very narrow range of foods [67]. Like neophobia, fussiness has been linked with decreased consumption of plant-based foods [68].

## 11. Early Feeding Practice

The role of breast-feeding on later dietary behaviour has been presented in a recent longitudinal study. Never being breast-fed or being breast-fed for a short duration was associated with lower healthy variety of food at 2, 3, and 4 years of age. On the contrary, there was no consistent association between the timing of complementary feeding and variety of diet, with a strong positive association between mother and children’s diet [69]. Breast-feeding has been proposed as an effective preventive intervention for low intake of vegetables in childhood [70]. Breast-feeding, compared with bottle-feeding, may promote maternal feeding styles that are less controlling and more responsive to infant cues of hunger and satiety, allowing infants greater self-regulation of energy intake [71].

Infant feeding practices are associated with later childhood dietary habits, but little is known about these relationships in non-Western countries with different food cultures. For this reason, Okubo et al. examined the association of breast-feeding duration and age at introduction of solid foods with later intake of fruit and vegetables in Japanese toddlers. Their findings were in line with those in Western countries: ≥6 months of breast-feeding may prevent low intake of vegetables in early childhood [72].

Baby-led weaning [BLW], where infants self-feed family foods in place of traditional weaning methods, is continuing to grow in popularity. In BLW, food is offered to the infant in whole form, as finger food, rather than puréed. Infants self-feed by selecting and grasping food, and join in family meals, consuming family foods [73].

A recent study demonstrates that infants weaned using a baby-led approach were significantly more satiety-responsive and less likely to be overweight, compared with those weaned using a standard approach [73]. This was independent of breast-feeding duration, timing of introduction to complementary foods, and maternal control. Mothers who adopt a BLW style are significantly different in personality, eating behaviour, and well-being characteristics. They exert significantly lower restraints and are less anxious compared with those utilizing the traditional approach. Parents who follow BLW report lower levels of restriction, pressure to eat, and monitoring of the child’s food intake [74]. Moreover, they are less concerned about the child’s body weight [74]. These characteristics may affect outcomes for infants weaned using this approach, but specific nutritional needs of infants should be considered during complementary feeding. Exclusion diets, which are increasingly common, may influence the behaviour of children too [75]. For instance, children consuming an exclusion diet for cows’ milk allergy have higher scores for feeding difficulties and fussy eating than those consuming an unrestricted diet, even if their growth is not affected [76]. Early-life experiences with healthy tastes and flavours may go a long way towards promoting healthy eating. This approach could have a significant impact in addressing the many chronic illnesses associated with poor food choices.

## 12. Obesogenic Environment

Childhood is a critical period in the development of obesity [77]. The feeding practices that evolved across human history as effective parental responses to the threat of food scarcity can, when combined with infants’ unlearned preferences and predispositions, promote overeating and overweight given the current availability of calorie-dense food [77]. Dietary behaviours thought to contribute to childhood obesity include appetitive traits such as: difficulty in matching the intake of energy to needs, a behaviour known as low responsiveness to internal satiety signals; high responsiveness to external food cues; high subjective reward experienced when eating liked foods; and preferences for energy-dense foods. All of these factors can influence the quantity of children’s food intake, and food preferences, which contribute to dietary ‘quality’ [75].

Modern diets based on unhealthy fast foods, convenience foods, energy-dense snacks, soft drinks, and on the abundance of food, sedentary lifestyles, and electronic recreation have led to serious overweight and obesity problems [75].

Parents who are overweight, who have problems controlling their own food intake, or who are concerned about their children’s risk for overweight may adopt controlling child-feeding practices in an attempt to prevent their children from becoming overweight too. Parents should be aware that large portions influence and promote total energy intake among children with poorer appetite regulation [78]. Accordingly, several studies demonstrated the effects of a larger package on increased total energy intake with a variety of foods, whereas reductions in portion or package size led to sustained decreases in energy intake [79].

When parents attempt to restrict their children’s eating to reduce weight and maintain health, emotional feeding, encouragement to eat, and fat restriction were associated with the development of obesogenic eating behaviours in children such as emotional eating, tendency to overeat, and food approach behaviours such as enjoyment of food and good appetite [80].

Overweight status and rapid weight gain during infancy are associated with increased fat mass, later risk of being overweight and numerous comorbidities. Savage et al., recently demonstrated that early responsive parenting [RP] intervention focused on infant soothing, sleeping, and feeding was associated with a significant decrease in infant weight gain and affected weight for length at age 1 year. In this Randomized Clinical Trial mothers received guidance on RP, which is defined as developmentally appropriate, prompt and contingent on their infant’s needs. RP promotes a range of adaptive outcomes in children including secure attachment, emotion regulation, cognitive and language development, and aspects of self-regulation including inhibitory control and executive function [81].

## 13. Media Influences

Cumulative exposure to television food advertising, which is higher in groups with lower SES, is linked to subsequent fast-food consumption in adults [82]. The media environment, and in particular commercials, have been shown to shape food-related knowledge, attitudes, preferences, and practices [82]. There is a direct causal link between advertising for food products and children’s diets; in particular, this occurs as an increase in snack food intake and overall calories and a decrease in consumption of fruits and vegetables [83]. Based on “choice context” in which children acquire their food knowledge, develop preferences, and actually make food choices, the need to create “junk-food-free environments” for children has gained increasing support from health professionals, consumer supporters, and concerned political groups. Children in Europe and the USA are heavily exposed to mass media. Depending on the children’s age and taking into account multiuse of media, recent reports show an average exposure of 8- to 18-year-olds in the USA to more than 7 h of electronic media per day [82]. As a result, in USA, foods consumed in front of the television account for about 20–25% of children’s daily energy intake [83]. In the IDEFICS study examining the effects of advertising on children’s food knowledge and preferences, as well as on dietary choices and weight status, pre-intervention adherence to some key behaviours related to childhood obesity, such as consumption of sweetened drinks and fruit/vegetables, daily television time, physical activity, family time, and adequate sleep duration, were evaluated. The adherence to international recommendations were converted into a composite score [83]. As adherence to the recommendations increased, a lower chance of being overweight/obese was observed. Overweight/obese children were more likely not to adhere to at least one of the recommended behaviours than normal-weight/thin children. The key findings of the study were that better food knowledge is not directly linked to healthier food preferences and that diet apparently has no significant effect on weight status [84,85]. This was thought to occur because the selected key behaviours do not contribute equally to a reduced chance of being overweight. Although consumer policy efforts to strengthen children’s ability to resist food industry lures have been tried, no effective “food marketing defence model” has been developed, and traditional policy strategies based primarily on informational and educational models are insufficient to decrease the effects of advertising on children. Parents and caregivers should be aware of their decisive role as choice planners and they should limit the exposure to television and time allotted to other sedentary behaviours.

## 14. Strategies to Improve Children’s Eating Behaviours

Feeding young children successfully requires some nutritional knowledge by the parent or caregiver to ensure that feeding practices and foods, as well as the amounts of calories offered, are appropriate. Intervention studies to identify ways to improve children’s eating behaviours are still very limited and strategies are age-related and not evidence-based. However, useful insights for parental practices can be extracted from cross-sectional and observational studies. Table 1 summarizes these strategies.

Researchers have found that children tend to require up to 15 exposures of a new food before it is “trusted” and thus tasted [28] and a further 10 to 15 exposures to bring about a liking of the food.

The likelihood that a given food will be offered is often linked to the likes and preferences of the parent. If parents themselves have a narrow diet, many foods will not appear on the table on enough occasions to allow for sufficient exposure [86] or for positive role modelling, which has been associated with increasing children’s acceptance of new foods and intake of healthier foods [87].

Other parenting practices can also have maladaptive influences on a child’s eating behaviour.

For example, giving foods, especially those high in fat or sugar, as reward, is a frequent parental practice. Past laboratory experiments show that repeatedly presenting snacks as a reward increases children’s preference for this kind of food. Food is a powerful reinforcement, and it maintains the behaviour on which its delivery or acquisition is dependent [23]. Giving a reward can result in increased preference for the reward food and decreased preference for the food that was initially promoted [21,79].

Moderation through control may be more effective than elimination or restriction of a highly palatable food. Pressure to eat has also been associated with increased food consumption. Overt restriction of foods has been associated with increased consumption of those foods when made freely available and greater weight gain over time [22].

Structure-based or limit-setting strategies, such as limiting how often certain foods are brought into the home and serving small portions, without forbidden access to these foods, provide children with opportunities to develop self-regulation and autonomy in eating behaviours [88]. Rollins et al. studied 180 mother-daughter dyads and measured the maternal reports of controlling feeding practices and girls’ height and weight, eating in the absence of hunger [EAH] at 5 years, and inhibitory control [a measure of behavioural inhibition] and approach [a measure of appetitive motivation] at 7 years. Their results showed that effects of the maternal inputs on the girls’ EAH and BMI may differ by the type of practice used [e.g., limit-setting or restrictive practices]. Girls with low inhibitory control were more susceptible to the negative effects of low and high control [88]. Moreover, even if restrictive feeding practices are counterproductive, and children with lower self-regulation and children at risk for obesity show greater susceptibility to the negative effects of restrictive feeding, some parental control is likely needed to moderate children’s intake of these foods [24].

Using food to soothe emotions and “make things better” has been found to be associated with increased BMI in 3–34-month-old children and eating more food in the absence of hunger in 3- to 4-year-old children, behaviours that have been linked to becoming overweight [88].

Parents have a difficult role: they are the example, they should model good habits, and pay attention to their own reaction towards food, so as to promote healthy food intake in their children and to bring only healthy food into the home. That is, the creation of a non-obesogenic child-rearing environment starts from sharing meals at home and creating a positive mealtime experience. Many authors suggest that parental controls should be avoided as they may potentially hinder the child’s capacity to develop adequate self-regulatory eating practices, which normally should be driven by hunger/satiety cues [20]. Nevertheless, Stifter et al. [88] conclude that, within an obesogenic society, a kind of moderate control is desirable.

## 15. Conclusions

Multiple factors influence dietary habits and are reciprocally interacting, so they cannot be viewed separately. The family system that surrounds a child’s domestic life will have an active role in establishing and promoting behaviours that will persist throughout his or her life. Fathers and mothers act differentially towards their children; fathers generally act in a more indulgent way and exert less active control on food intake. In an obesogenic environment, authoritative behaviour and some parental control is likely needed to moderate children’s intake of palatable calorie-dense foods. Limiting how often certain foods are brought into the home environment, avoiding stores and restaurants that sell unhealthy foods, and serving small but adequate portions should provide children with opportunities to develop self-regulation in eating behaviours. Early-life experiences with various tastes and flavours have a role in promoting healthy eating and favouring wider consumption of fruits and vegetables. Offering infants different foods beginning in the complementary feeding period and providing repeated exposure of disliked foods to stimulate their taste and help them to accept many foods later in life is a necessary strategy to develop good eating habits. All of these strategies come together during family meals. This setting has significant social importance in a child’s life and parents should expose their offspring to a range of good food choices while acting as positive role models to shield children and adolescents from the hazards of the obesogenic environment of modern life. Socioeconomic status is involved in these issues, as families where the parents have high educational levels consume more healthy foods than other families who are less aware of the issues. Accordingly, educational programmes should be offered to all children from different socioeconomic levels, with a goal of promoting physical activity, reducing television, video game, and computer time, and getting adequate sleep. Parents should receive advice on how to establish long-term healthy habits and to create pleasant eating patterns in their children, while becoming aware of behavioural determinants that favour malnutrition and eating disorders.

## Figures and Tables

**Table 1:** Strategies to improve child’s eating behaviour.

Strategy	Practices
Covert control	- Purchasing only healthy foods at home - Avoidance of unhealthy stores and fast food
Avoid the use of food rewards	- Food maintains the behaviour on which its delivery and acquisition is dependent
Promoting self-regulation	- Recognition of fullness sense - Serving moderate portions - Help in organizing the feeding environment
Authoritative parenting style	- Encourage children to try new foods - Parents are the example - Parent models healthy eating and enjoyment of foods - Do not model disliking of foods in front of child - In obesogenic environment, some parental control is likely needed to moderate children’s intake of palatable snack foods - Early responsive parenting [RP] intervention
Family meals	- Expose to a variety of foods - Repeatedly expose child to a food - Allow child to have input into food choices - High frequency of shared family meals - Daily shared breakfast - Socialization during mealtime - Turn off TV at meals
Parent’s focused intervention	- Educationally-based interventions adapted to parents and caregivers - Feeding-related advice - Empowering parents - Social support
Family environment	- Early-life experiences with healthy tastes and flavours may promote healthy eating - Give the parental role in food shopping and preparation - Healthy food availability - Reduce screen time and get adequate sleep
